# Microcystin-LR Does Not Alter Cell Survival and Intracellular Signaling in Human Bronchial Epithelial Cells

**DOI:** 10.3390/toxins12030165

**Published:** 2020-03-07

**Authors:** Ondřej Brózman, Barbara Kubickova, Pavel Babica, Petra Laboha

**Affiliations:** 1 RECETOX, Faculty of Science, Masaryk University, Brno 62500, Czech Republic; ondrej.brozman@recetox.muni.cz (O.B.); barbara.kubickova@recetox.muni.cz (B.K.); pavel.babica@recetox.muni.cz (P.B.); 2 Department of Experimental Phycology and Ecotoxicology, Institute of Botany, Czech Academy of Sciences, Brno 60200, Czech Republic

**Keywords:** microcystin-LR, human bronchial epithelial cells, in vitro, HBE1, 16HBE14o-, mitogen-activated protein kinase, cytotoxicity, OATP

## Abstract

Changes in ecological and environmental factors lead to an increased occurrence of cyanobacterial water blooms, while secondary metabolites-producing cyanobacteria pose a threat to both environmental and human health. Apart from oral and dermal exposure, humans may be exposed via inhalation and/or swallowing of contaminated water and aerosols. Although many studies deal with liver toxicity, less information about the effects in the respiratory system is available. We investigated the effects of a prevalent cyanotoxin, microcystin-LR (MC-LR), using respiratory system-relevant human bronchial epithelial (HBE) cells. The expression of specific organic-anion-transporting polypeptides was evaluated, and the western blot analysis revealed the formation and accumulation of MC-LR protein adducts in exposed cells. However, MC-LR up to 20 μM neither caused significant cytotoxic effects according to multiple viability endpoints after 48-h exposure, nor reduced impedance (cell layer integrity) over 96 h. Time-dependent increase of putative MC-LR adducts with protein phosphatases was not associated with activation of mitogen-activated protein kinases ERK1/2 and p38 during 48-h exposure in HBE cells. Future studies addressing human health risks associated with inhalation of toxic cyanobacteria and cyanotoxins should focus on complex environmental samples of cyanobacterial blooms and alterations of additional non-cytotoxic endpoints while adopting more advanced in vitro models.

## 1. Introduction

Cyanobacteria, the most diverse group of Gram-negative prokaryotes and Earth’s oldest known oxygen photoautotrophs, are an important part of both terrestrial and aquatic ecosystems [1,2,3]. Cyanobacteria are experiencing a boom in recent years along with the increasing eutrophication of the environment, decreased diversity of phytoplankton, rising CO_2_ levels, and global increase of temperature [4,5,6,7]. Nowadays, the frequent and often massive occurrence of cyanobacteria-dominated water blooms associated with the production and release of a large variety of biologically active secondary metabolites, cyanobacterial toxins (cyanotoxins), has become a major environmental and health problem [8,9]. The variety of toxic effects linked to cyanobacterial blooms ranges from dermal irritation, gastrointestinal symptoms, inflammation, hepatotoxic, nephrotoxic, neurotoxic effects, to neoplastic transformation and cancer [4].

Humans can be exposed to cyanobacterial toxins via drinking of water, consumption of contaminated food, or during recreational activities when contaminated water is swallowed or inhaled [2,4,8]. Whilst oral and dermal exposures are considered the main routes of cyanotoxins entering the human body, the inhalation exposure has gained attention relatively recently [10], along with the fact that aerosolized cyanobacteria have been detected in the human upper respiratory tract and central airways [11] and several toxin-producing cyanobacteria including *Microcystis* sp. were found in the aerosol samples [12]. Cyanobacteria and associated toxins may enter the human body through inhalation of aerosolized particles from wave breaking [12,13] or inhalation/swallowing of contaminated water during swimming and other recreational activities, such as paddling or surfing [4,14].

Microcystins (MCs) are an environmentally abundant class of cyanotoxins [1,4]. MCs are a large group of monocyclic non-ribosomal heptapeptide toxins [15], varying primarily in their two L-amino-acids. These toxins can be produced by terrestrial cyanobacterial genera, such as *Hapalosiphon*, as well as by both bloom-forming marine and freshwater cyanobacteria, including the genera *Microcystis*, *Planktothrix*, or *Dolichospermum* [3,4,16]. MCs are transported via blood and bile carriers into target organs such as the liver, intestine, kidneys, and lungs [8]. Several human and animal intoxications by MC-producing cyanobacteria have been recorded following multiple exposure routes, including inhalation, as thoroughly reviewed in Svirčev et al. [17]. Overall, the collected data suggest that the mammalian respiratory system is susceptible to MCs regardless of the exposure route [18].

Over 270 different structural analogs of MCs with varying toxicity to mammals were found so far [17,19], among which, microcystin-LR (MC-LR) is the most abundant and widely studied variant [2,20]. MC-LR is a heptapeptide containing L-leucine (L) and L-arginine (R) in positions 2 and 4 within its structure [16]. Due to their hydrophilic character and the relatively high molecular mass (approx. 1 kDa) in comparison to freely diffusible ions and small organic compounds, the absorption and cellular uptake of MC-LR is facilitated by organic-anion-transporting polypeptides (OATP) present in a majority of human organs and tissues, rather than by passive diffusion [21,22]. 

MC-LR is considered to be a tumor promoter [2]. According to the statement of the International Agency for Research on Cancer (IARC), MC-LR has been designated as “possibly carcinogenic to humans”, group 2B [23]. Main mechanisms of action include impairment of intracellular phosphorylation processes caused by dose-dependent inhibition of serine/threonine protein-phosphatases (PP), especially PP1 and PP2A [9,21,24]. PPs counteract diverse intracellular kinases such as Akt, mitogen-activated protein kinases (MAPKs), protein kinases (PK) A and C, thus are responsible for maintaining multiple vital processes such as cell cycle, cytoskeleton organization, cell proliferation, apoptosis, migration, mobility, and survival [4,9,25]. MC-LR exposures have been linked to genotoxicity and tumor promotion [4,26], both induction of cell growth and increase in apoptosis depending on a dose [27], reactive oxygen species (ROS) production leading to oxidative stress [28] and impaired function of mitochondrial DNA [29], immunotoxicity [30], altered immune responses [31], toxicity to reproductive organs [32], neurotoxicity [33], neoplastic transformation, and transformed phenotype in cancer and lung carcinoma [34]. 

In general, human exposure to cyanotoxins, including MC-LR, may lead to both acute and chronic effects [3]. Chronic exposure to MC-LR results in sustained PP inhibition with subsequent hyperphosphorylation of intracellular proteins, such as MAPKs (e.g., extracellular signal-regulated kinases 1/2, ERK1/2), changes in oncogenes expression and TNF-α expression [5]. An increased incidence of colorectal and hepatic cancers is associated with chronic exposure to MCs [35]. Acute effects involve changes in cell morphology, oxidative stress (formation of ROS and/or glutathione depletion), disruption of actin in intermediate filaments, altered expression of pro-apoptotic proteins, mitochondrial damage, and defects in cell adhesion [9,17,36]. 

Although there are many studies about liver toxicity and associated adverse effects, distinctly less information about the effects of MCs in the respiratory system is available. The observed effects and findings related to MC-LR exposure affecting the respiratory tract are summarized in Table 1.

Additionally, Wang et al. [45] observed decreased levels of cytoskeletal components leading to alterations in cell–cell communication in a dose-dependent manner, increased activation of MAPK-ERK1/2 and Akt, but no significant changes in inflammatory markers (IL-1, IL-6, nor TNF-α) in the mice alveolar type II epithelial cells. The relevance and importance to study the effects of MC-LR in the lungs is underlined by the fact that MC-LR influences tight junction proteins that play an important role in the alveolar epithelial barrier function [46,47]. It was documented that in vitro exposure of human bronchial epithelial cells to MC-LR resulted in decreased viability, determined by tetrazolium reduction assay, in a concentration-dependent manner [29]. Another study demonstrated that MC-LR is capable to inhibit PP2A, activate MAPK pathways (p38, ERK1/2) and anti-apoptotic genes (Akt, B-cell lymphoma 2), and induce cytoskeletal changes, but not to interfere with either apoptosis or proliferation in A549 human non-small lung cancer cells after 24-h exposure [25]. These findings suggest that MC-LR causes adverse effects in both lung tissue and respiratory system-relevant in vitro systems. 

To address the data gap in the molecular mechanism and uptake into human bronchial epithelia, we utilized two immortalized human bronchial epithelial cell lines (HBE1 and 16HBE14o-) and studied the effects of MC-LR on a (sub)cellular level. We investigated the MC-LR uptake into bronchial cells and evaluated the effects on in vitro cell growth and the ability to interfere with cell signaling pathways.

## 2. Results

### 2.1. MC-LR Uptake and The Expression of Genes Encoding OATPs

**Figure 1:**
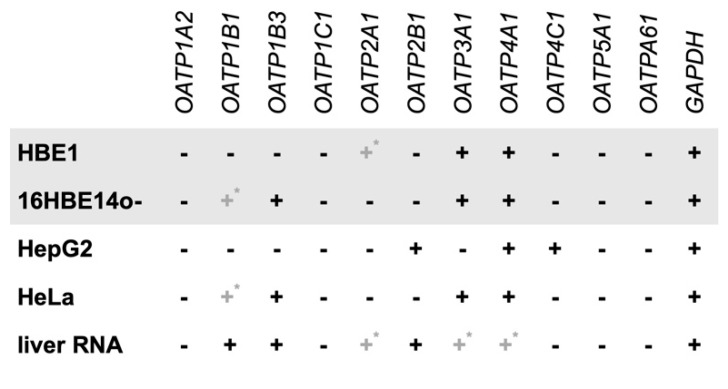
Expression of genes encoding Organic-Anion-Transporting Polypeptides (OATPs) in HBE1 and 16HBE14o-cells. RT-PCR was used to assess the expression of specific OATP isoforms in both HBE1 and 16HBE14o-cell lines. HepG2, human hepatocellular carcinoma cells, HeLa, human epithelial cervical adenocarcinoma cells, and liver RNA were used as a positive control. Plus sign (+) represents positive detection, dash sign (−) represents an absence of the specific polypeptide, grey plus sign with asterisk (+*) indicates weak expression. *GAPDH*, glyceraldehyde-3-phosphate dehydrogenase; *OATP*, organic-anion-transporting polypeptide.

Firstly, we evaluated whether both HBE1 and 16HBE14o- cells express genes encoding OATPs that are responsible for MC-LR cellular uptake. Reverse-transcription polymerase chain reaction (RT-PCR) was used to determine the presence or absence of eleven individual OATP isoforms in both HBE1 and 16HBE14o- cells. The experiments were performed in parallel with HepG2 cells, HeLa cells, and liver RNA, which served as control samples (Figure 1). Representative images of gene expression detected by RT-PCR are shown in the Supplementary Materials (Figure S1).

Our results show that both studied bronchial cell lines express multiple OATP-encoding genes, including OATP3A1 and 4A1 isoforms (Figure 1). Further, the OATP1B3-encoding gene was expressed in 16HBE14o-cell line, along with a weak expression of OATP1B1, while neither OATP1B1 nor 1B3 transcripts were detected in HBE1. On the other hand, less pronounced expression of the OATP2A1-encoding gene was detected only in HBE1 cells. We demonstrate that both HBE1 and 16HBE14o- express at least two genes encoding OATPs, in case of 16HBE14o- isoforms from family 1 known to facilitate MC-LR transport, OATP1B1, and 1B3.

Further, the uptake of MC-LR by human bronchial cells was indicated by western blotting (Supplementary Materials, Figure S2). We observed the formation of bands recognized by anti-MC-LR antibody in both HBE1 as well as 16HBE14o- cells. These bands were detected only in MC-LR exposed cells, and their intensity was increasing over 48 h of exposure, which indicates that these bands represent adducts of MC-LR with cellular proteins that accumulate in the exposed bronchial cells.

### 2.2. Viability Assays

**Figure 2:**
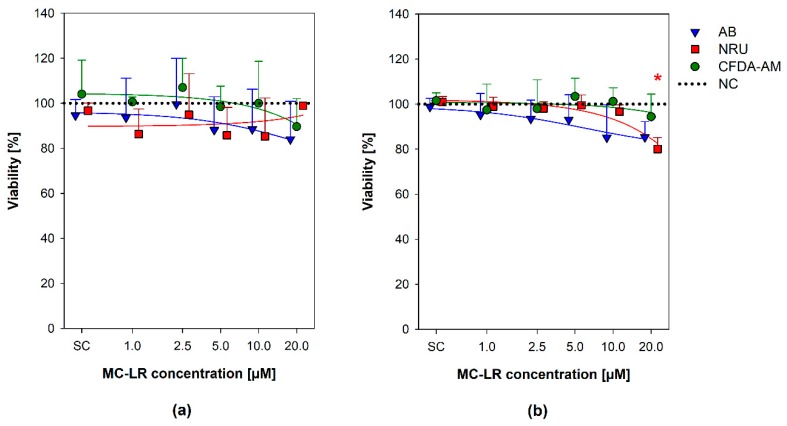
Effects of microcystin-LR (MC-LR) on the viability of HBE1 and 16HBE14o- cells. A combination of three assays was adopted to assess the viability of HBE1 cells (**a**) and 16HBE14o- (**b**) exposed to 1, 2.5, 5, 10, and 20 µM MC-LR for 48 h. Values are plotted as a percentage of viability in comparison with the negative control (NC; untreated, 100% viability, indicated by the dotted line). The scatter plot represents mean + S.D. from independent experiments (n ≥ 3) conducted in triplicate. Asterisks (*) indicate values significantly different from the negative control (ANOVA followed by Dunnett’s *post-hoc* test, *p* ˂ 0.05). *SC*, solvent control; *CFDA-AM*, 5-carboxyfluorescein diacetate acetoxymethyl ester; *AB*, Alamar blue®; *NRU*, neutral red uptake; *NC*, negative control; *MC-LR*, microcystin-LR.

The effects of MC-LR on viability of both HBE1 and 16HBE14o- cells were evaluated using a combination of three endpoints based on cell metabolic activity, namely plasma membrane integrity and esterase activity assessed by CFDA-AM, metabolic reductive potential (Alamar blue® assay), and neutral red uptake (NRU) by lysosomes (Figure 2) [48,49]. 

The tested MC-LR concentrations (1–20 µM MC-LR) caused none or only minor cell viability decreases in both HBE1 and 16HBE14o- cells in all the three CFDA-AM, AB, and NRU cytotoxicity assays (Figure 2). No apparent concentration-response trend was found for HBE1 cells (Figure 2a), where all the viability results were comparable to the level of negative (naïve) control (NC; cells grown in culture medium without any treatment) and not significantly different. In 16HBE14o- cells, a slight gradual decrease in the viability was observed with an increasing MC-LR concentration in all three endpoints (Figure 2b), with a statistically significant decrease of NRU (down to ~80% viability compared to NC) observed after exposure to 20 µM MC-LR. 

### 2.3. Real-Time Cell Analysis 

**Figure 3:**
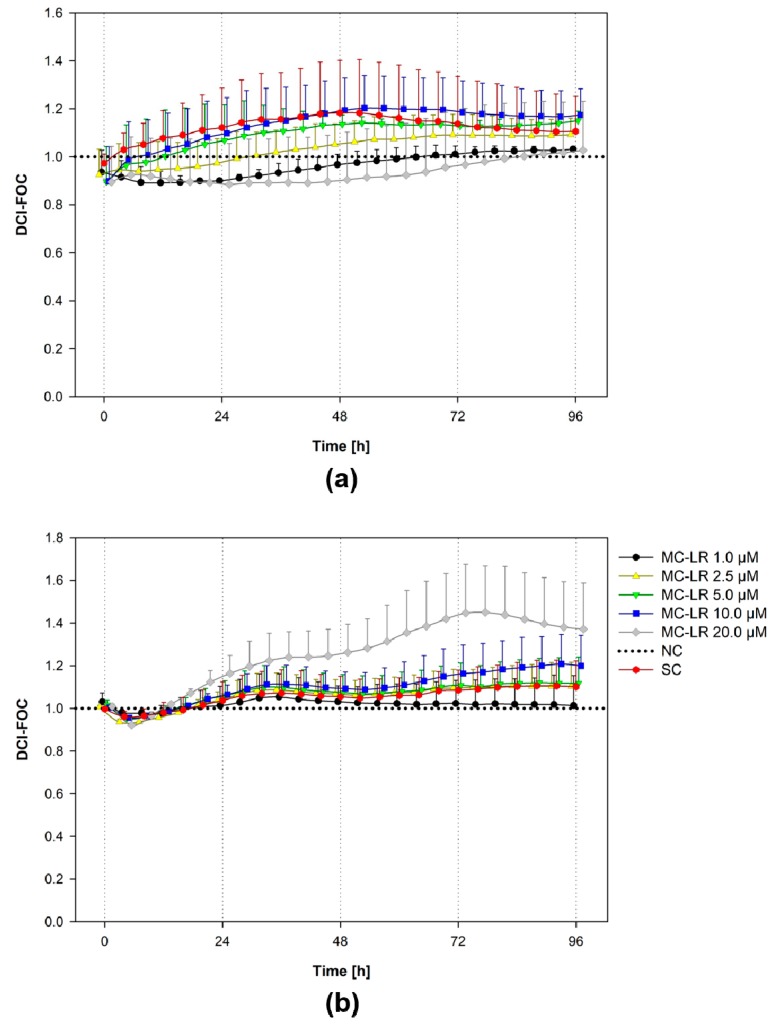
Effects of microcystin-LR (MC-LR) on the impedance induction by HBE1 and 16HBE14o- cells. In the real-time cell analysis experiments, HBE1 cells (**a**) and 16HBE14o-cells (**b**) were grown in culture medium for 24 h and then exposed to 1, 2.5, 5, 10, and 20 µM MC-LR for 96 h. Values are plotted as Delta Cell Index-Fraction of the non-treated Control (DCI-FOC), data normalized according to Equation S1 in the Supplementary Materials. The scatter plot represents mean + S.D. from two independent experiments conducted in duplicate. *SC*, solvent control; *NC*, negative control; *MC-LR*, microcystin-LR.

To further increase the knowledge about the effects of MC-LR (1–20 µM) on bronchial cell viability, we conducted real-time cell analysis (RTCA) experiments with continuous 96-h measurement of the cell electrical impedance, which reflects cell viability, proliferation, and also monolayer cohesion/adhesion (Figure 3). 

In general, there were only minor alterations in response to MC-LR treatment observed by RTCA. HBE1 cultures demonstrated no apparent trends in cell impedance (Figure 3a). Indeed, HBE1 cell impedance was relatively low compared to 16HBE14o-, suggesting that HBE1 did not form a tight epithelial monolayer. 

The impedance of 16HBE14o- cultures treated with 1-5 µM MC-LR closely corresponded to the solvent control (SC; Figure 3b). The exposure to 10 µM MC-LR resulted in a minor impedance increase compared to both NC and SC. The increase in cell impedance was more pronounced after 20 µM MC-LR treatment, but not statistically significant (ANOVA followed by Dunnett’s *post-hoc* test, *p* ˂ 0.05). 

### 2.4. Evaluation of MAPKs Activity

Effects of MC-LR exposure on the activation (phosphorylation) of cellular MAPKs ERK1/2 and p38 kinases were investigated. Bronchial epithelial cells were exposed in time-lapse experiments (0.1, 1, 2, 8, 24, and 48 h) to a non-cytotoxic and toxicologically-relevant concentration of 1µM MC-LR, which was shown to form MC-LR protein adducts in both cell lines (see the Supplementary Materials, Figure S2). Proteins isolated from both HBE1 and 16HBE14o- cells were examined using western blotting technique. Both phosphorylated and total ERK1/2 (P-ERK1/2 and t-ERK1/2) were detected as a major band with a molecular weight of 42 kDa and a faint band of 44 kDa. Phosphorylated and total p38 (P-p38 and t-p38) were detected as a distinct band at 38 kDa. All experiments were carried out at least in two biological replicates together with untreated negative control (NC) and appropriate solvent control (0.04% (*v*/*v*) methanol). For detailed calculation procedures see the Supplementary Materials, Equation S2.

**Figure 4:**
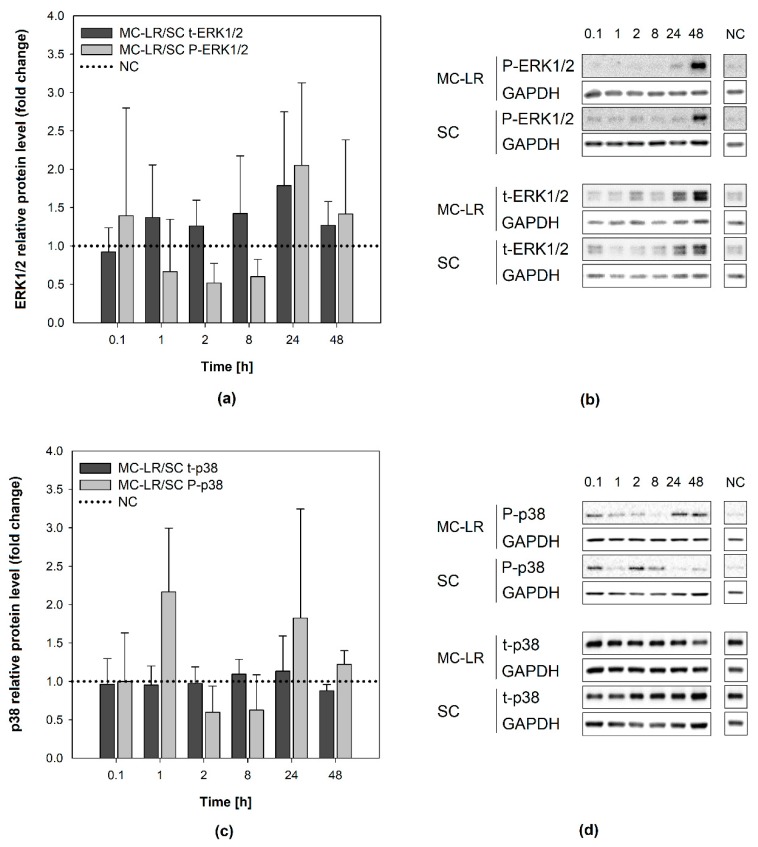
Effects of 1 µM microcystin-LR (MC-LR) on mitogen-activated protein kinases (MAPKs) activation in HBE1 cells. Results of densitometric evaluation show MAPK activation (phosphorylation) by MC-LR treatment expressed as fold changes over the solvent control (SC) with normalization to the negative control (NC; horizontal dotted line): fold changes in (**a**,**b**) extracellular signal-regulated kinases 1/2 (ERK1/2) and (**c**,**d**) p38 kinase activation. Data were normalized according to Equation S2 in the Supplementary Materials. Bar charts represent mean + S.D. from independent experiments (n ≥ 2). *SC*, solvent control; *MC-LR*, microcystin-LR; *NC*, negative control; *P-ERK1/2*, phosphorylated extracellular signal-regulated kinases 1/2; *t-ERK1/2*, total extracellular signal-regulated kinases 1/2; *P-p38*, phosphorylated p38 kinase; *t-p38*, total p38 kinase; *MC-LR/SC*, ratio between MC-LR and SC densitometric values; *GAPDH*, glyceraldehyde-3-phosphate dehydrogenase.

In HBE1 cells, the levels of either P-ERK1/2 or t-ERK1/2 following MC-LR exposure were not statistically significantly different (t-test, *p* > 0.05) from SC (Figure 4a,b). Although the P-ERK1/2 levels were elevated following 48-h MC-LR exposure, this phenomenon was observed also in the solvent control (Supplementary Materials, Figure S3a). Hence, there was no significant change in P-ERK1/2 levels after MC-LR treatment in comparison to the corresponding SC treatment (t-test). The levels of total ERK1/2 were not altered in MC-LR treatments or SC, and not significantly different from the NC (Supplementary Materials, Figure S3b). Similarly, no significant p38 activation by MC-LR was observed (Figure 4c,d), since P-p38 levels were elevated to a similar extent in both MC-LR and SC treatment after 48 h (~11x and ~9x NC, respectively). Total p38 levels remained at the level of NC throughout the experiment (Supplementary Materials, Figure S3c,d). 

**Figure 5:**
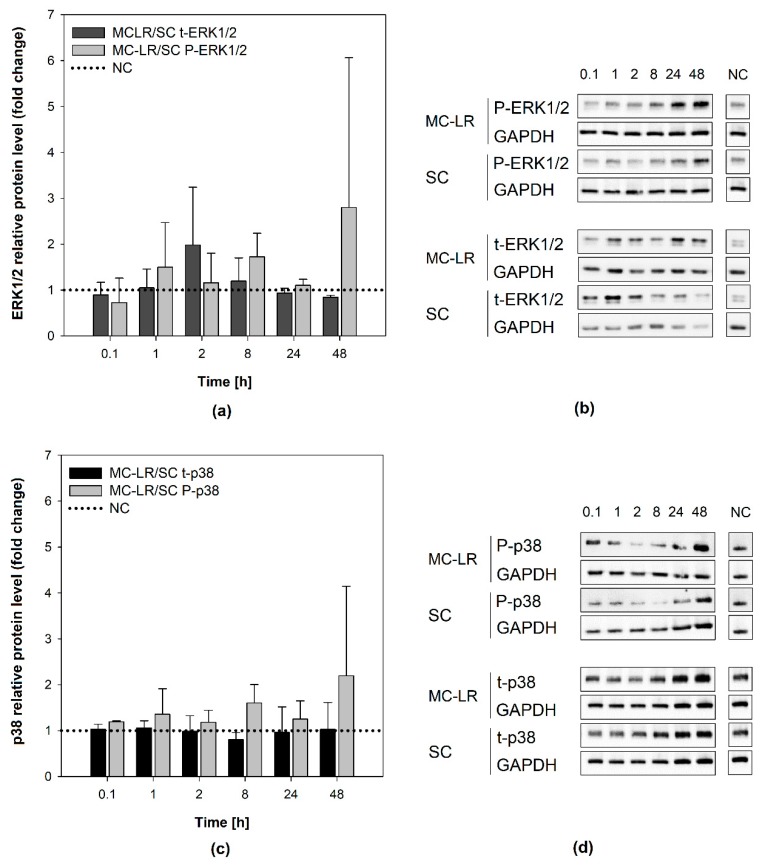
Effects of 1 µM microcystin-LR (MC-LR) on mitogen-activated protein kinases (MAPKs) activation in 16HBE14o- cells. Results of densitometric evaluation show MAPK activation (phosphorylation) by MC-LR treatment expressed as fold changes over the solvent control with normalization to the negative control (horizontal dotted line): fold changes in (**a**,**b**) extracellular signal-regulated kinases 1/2 (ERK1/2) and (**c**,**d**) p38 kinase activation. Data were normalized according to Equation S2 in the Supplementary Materials. Bar charts represent mean + S.D. from independent experiments (n ≥ 2). *SC*, solvent control; *MC-LR*, microcystin-LR; *NC*, negative control; *P-ERK1/2*, phosphorylated extracellular signal-regulated kinases 1/2; *t-ERK1/2*, total extracellular signal-regulated kinases 1/2; *P-p38*, phosphorylated p38 kinase; *t-p38*, total p38 kinase; *MC-LR/SC*, ratio between MC-LR and SC densitometric values; *GAPDH*, glyceraldehyde-3-phosphate dehydrogenase.

Similarly, no significant increase of P-ERK1/2 levels was observed in 16HBE14o- cells following 48-h exposure to 1 µM MC-LR (Figure 5a,b). Total ERK1/2 levels in MC-LR treated cells remained stable throughout the experiment and comparable to SC and NC (Supplementary Materials, Figure S4a,b). Slight time-dependent increase in p38 activation after 48-h MC-LR exposure was not significantly different from SC (Figure 5c,d). Total levels of p38 remained unaltered by MC-LR during the experiments in all experimental variants (Supplementary Materials, Figure S4c,d). Overall, the results show no major changes in the activation of ERK1/2 and p38 caused by 1 µM MC-LR in HBE1 and 16HBE14o-cells, suggesting that MC-LR does not significantly interfere with intracellular signaling in these cells. 

## 3. Discussion

The increasing abundance and frequency of harmful algal and cyanobacterial blooms is gaining public attention in recent years since it is negatively influencing recreational activities in many freshwater and coastal areas. The research has been recently oriented towards hazards resulting from inhalation exposure to cyanobacterial aerosols and toxins. To evaluate possible effects of a very abundant cyanotoxin MC-LR in the human respiratory system, we used two immortalized, non-cancerous, respiratory-relevant human bronchial epithelial cell lines. The concentrations of MC-LR in our experiments were chosen based on previous studies with human bronchial or alveolar cells [25,29], where MC-LR at relatively high concentrations (30–40 µM) nearly completely inhibited cell viability after 24-h exposure, while 1–20 µM significantly reduced cell viability to 90%–35% of the control [29]. Thus, in our prolonged exposure (48–96 h) experiments, we decided to test a concentration range of 1–20 µM MC-LR, while using 1 µM MC-LR to assess the effects of a toxicologically more feasible concentration on a sub-cellular level for mechanistic studies.

First, we determined the transcription of specific OATP-encoding genes by reverse-transcription polymerase chain reaction (RT-PCR) in order to see if the toxin can be potentially transported into these cells to further manifest both cellular and subcellular effects. Out of 11 different human OATP isoforms, the ability to transport MC-LR was confirmed for OATP family 1 (OATP1A2, 1B1 and 1B3), while no MC-LR transport was detected for the OATP2B1 [50]. To the best of our knowledge, the other OATP isoforms have not been investigated for their ability to transport MC-LR [36]. Regarding OATP expression in human lung tissue, studies utilizing microarray and RT-PCR reported expression of OATP2B1, 3A1, 4A1, and 4C1 [51,52], while targeted proteomics analyses detected OATP1A2, 1B3, 2A1 and 2B1 [53]. In vitro, OATP3A1 and 4A1 were found by PCR to be consistently expressed in various types of human bronchial and alveolar cells, including primary bronchial epithelial cells and immortalized cell lines such as Calu-3, Beas-2B, 16HBE14o-, and A549 [54,55], which is consistent also with our results for both, 16HBE14o- and HBE1 cell lines. 

However, expression of other OATP isoforms, including MC-LR-transporting OATP1A2, 1B1, and 1B3, seems to be varying between different in vitro models and culture conditions [54,55]. Expression of OATP1A2 was reported in primary bronchial epithelial cells, cell lines Calu-3, Beas-2B, and A549, but not 16HBE14o-, which is also in agreement with our results. OATP1B1 and/or 1B3 were previously detected in Calu-3, Beas-2B or A549, but not in primary bronchial epithelial cells or 16HBE14o- [54,55]. Interestingly, we found OATP1B1/1B3 genes to be transcribed under our experimental conditions by the 16HBE14o-, but not by the HBE1 cell line.

Nevertheless, the formation of MC-LR protein adducts in the exposed cells was qualitatively and quantitatively comparable between both HBE1 and 16HBE14o- cell lines, which indicates that other OATP isoforms than 1B1/1B3 (e.g., 3A1 or 4A1), or other membrane transporters or cellular mechanisms, could have been involved in the uptake of MC-LR by bronchial epithelial cells. This is consistent with a previous study, which reported formation of MC-LR protein adducts in immortalized adult human liver cells HL1-hT1 occurring independently on the expression or activity of OATP1B1/1B3, since HL1-hT1 cultures were found to express OATP2A1 and 3A1 only, and the putative MC-LR uptake was not affected by pharmacological inhibitors of OATP1B1/1B3 [56]. MC-LR-protein adducts accumulated in both HBE1 and 16HBE14o- cells included major bands with molecular weights around 35–37 kDa, i.e., corresponding to PP1/PP2A catalytic subunits, which represent primary intracellular targets known to covalently bind MC-LR [57]. Additional protein bands detected by anti-MC-LR antibody had molecular weights around 20 kDa, 30 kDa, and in the case of 16HBE14o- cells also 60 kDa, which might correspond to putative intracellular targets of MC-LR (e.g., 55 kDa ATP-synthase β subunit, [58]; 56 kDa aldehyde dehydrogenase 2 [59]; or 23 kDa Proteasome β2 subunit [60], or to yet-to-be-identified proteins [56]). 

Although these results suggest uptake of MC-LR and its interaction with cellular proteins, the viability assays did not reveal any major effects of MC-LR in both HBE1 and 16HBE14o-cells. The results of our study are mostly consistent with findings of Wang et al. [25], who found no significant effect of 10 µM MC-LR on viability in A549 human non-small lung cancer cells following 24-h exposure. On the contrary, Li et al. [29] found that 10 µM MC-LR significantly reduced (EC50) cell survival in HBE cells after 24 h, and concentrations ≥50 nM were cytotoxic to immortalized murine alveolar type II cell line [45]. To compare with other epithelial cell lines, a decreased viability of rat Sertoli cells was found after 24-h exposure to 8–32 µM MC-LR [61]. Additionally, a time-dependent reduction in survival was observed in a series of murine RAW246.7 macrophage-like, BV-2 immortalized microglial, and N2a neuroblastoma-derived cells exposed to 10 µM MC-LR for 24–72 h [62]. However, 10 µM MC-LR did not decrease the viability of human adult liver stem cells HL1-hT1 [56,63], human hepatocellular carcinoma cells HepG2, human colorectal carcinoma cells Caco-2 or monkey kidney epithelial Vero-E6 line [64,65,66]. The data presented in the literature thus suggests that the cytotoxic effects of MC-LR are cell type-specific and possibly species-specific. 

Impedimetric RTCA revealed no major changes in cell adhesion and proliferation rates of HBE1 cells. Although both HBE1 and 16HBE14o- cells reached relatively stable impedimetric values 24 h post-seeding, HBE1 elicited lower impedance than 16HBE14o-, similarly to previous observations (see Figure 2a in [67]). An impedance increase (~35%) following 20 µM MC-LR exposure of 16HBE14o- cells was not accompanied by an increase of metabolic activity but rather by a slight decrease (see AB, CFDA-AM, and NRU results). A similar trend was observed in 16HBE14o- cultures exposed to lower concentrations of cytotoxic cyanotoxin cylindrospermopsin (0.5 µM CYN), whilst an initial impedance increase observed in the 1–2.5 µM CYN treatment for approximately 60–90 h was followed by a steep decline of the cell impedance and accompanied by a decrease of cell viability [67]. Further, Basu et al. [63] found that 10 µM MC-LR induced a slight (∼20%) increase in cell impedance of the adult human liver stem cells HL1-hT1 in monolayer experiments that was not accompanied by an increase of metabolic activity.

The formation of in vivo barriers between different compartments of the body as well as pseudo-epithelial barriers in vitro requires the formation of tight junctions including the tight junction protein ZO1/TJP (reviewed in [68]). ZO1/TJP protein was present in 16HBE14o- cells, indicating the formation of tight junctions and a tight (pseudo-)epithelial sheet, supported by the measurement of transepithelial resistance (TEER) [67]. However, no ZO1/TJP and very low values of TEER measurement were recorded for HBE1 cells, thus suggesting that HBE1 cultures were not able to form tight junctions [67]. This might explain the lower ability of HBE1 cells to induce cell impedance and its measurable changes. 

Tight junctions work as paracellular gates that restrict diffusion on the basis of size and charge. Such selective paracellular diffusion is essential to maintain homeostasis in organs and tissues [68]. Based on the aforementioned results and studies, we can speculate that 16HBE14o- cultures react to the presence of cyanotoxins (at a certain concentration level) via an increase in impedance in order to tighten the paracellular diffusion as a part of the adaptive stress response. Such an effect of epithelial cells in the respiratory tract is desired in order to restrict transfer of bigger molecules, including cyanotoxins, further into the body. However, the toxicological tipping point of the adaptive stress response may be surpassed with further cyanotoxin concentration increase and adverse effects on bronchial epithelial layers might be expected, as shown for CYN [67]. In addition, MC-LR-induced reduction of viability in murine epithelial alveolar type II (ATII) cells was accompanied by a dose-dependent decrease of TEER values and reduced expression of tight junction proteins (e.g., ZO-1, occludin) [45]. MC-LR also decreased the expression of tight junction proteins in murine Sertoli cells [69]. Therefore, further studies, preferably with physiologically more relevant human in vitro models, such as filter-insert cultures or 3D air-liquid interface (ALI) cultures of bronchial epithelial cells [70] should focus on tight junctions and barrier function modulation in response to cyanotoxins, especially in combination with relevant exposure estimates and scenarios. 

Intracellular signaling pathways, such as ERK1/2, p38, and JNK signaling, play a critical role in the control of inflammatory respiratory diseases and cell proliferation-related neoplastic disorders [71]. Disruption of MAPK signaling by MC-LR has been documented in several studies with multiple in vitro models [24,25,26,45,72] and might be a direct consequence of MC-LR-induced inhibition of PP1/PP2A. Although the MC-LR-positive protein bands were formed and accumulated in both HBE1 and 16HBE14o- cells, we observed only minor changes in the activation of ERK1/2 and p38 kinases, suggesting that non-cytotoxic concentrations of MC-LR did not significantly interfere with intracellular MAPK signaling in these cells. Similarly to cell viability or barrier function, responses and sensitivity of MAPKs to MC-LR exposure seem to depend on the cell type and in vitro model used. For example, Wang et al. [45] observed significantly increased ERK1/2 activity after 24-h exposure to 0.5 µM MC-LR in murine epithelial ATII cells; however, Wang et al. [25] reported no significant changes in P-ERK1/2 following 24-h exposure to 1 µM MC-LR in human A549 lung cancer cells. Increased phosphorylation was observed in the human HL7702 liver cell line after 48-h exposure to 10 µM MC-LR [72], but not in human HL1-hT1 adult liver stem cells exposed to 1 µM [56], despite toxin uptake was documented in both studies. Elevated P-ERK1/2 levels were additionally reported by Adamovsky et al. [31] in murine RAW 264.7 macrophages after 30-min exposure to 1 µM MC-LR, but probably triggered via interactions of the toxin with membrane receptors, independently of MC-LR cellular uptake and inhibition of PPs.

Overall, our observations add further evidence about cell line-, tissue of origin-, and even species-specificity of MC-LR effects. The use of relevant in vitro models providing the desired biological context in terms of target species, tissue, and cell type is, therefore, necessary for accurate identification of human health hazards and assessment of risks, including the risks associated with the inhalation of aerosolized cyanotoxins, such as MC-LR. Thus, our experiments with immortalized, non-cancerous human bronchial epithelial cells provide valuable insight into potential effects induced in the human airway epithelium exposed to MC-LR. Despite the absence of stronger cytotoxic responses or detectable disruptions of MAPK signal transduction, the toxin was apparently accumulated by the cells and interacted with several cellular proteins. While MC-LR represents the most studied structural variant among MCs because of its high acute hepatotoxicity and frequent environmental occurrence, cyanobacterial blooms frequently contain multiple MC variants at the same time, some of them demonstrating even higher toxicity and cell permeability than MC-LR [21,22,73,74].

Considering environmental concentrations of MCs, these toxins can be occasionally detected in surface waters at levels up to several mg/L (corresponding to several µM), but typical concentrations are usually within the range of several µg/L (~nM) [4]. For example, a recent survey mapping of cyanotoxin concentrations in 137 European lakes detected MCs (sum of different variants) in 93% lakes with a mean concentration of 1.2 µg/L (~1.2 nM), and a maximum of 17.2 µg/L (~17.2 nM) [75]. A first guideline value recommendation of 20 µg/L for moderate risk of adverse effects due to MC exposure in recreational waters was given by the WHO in 2003 [76] and refined by the US Environmental Protection Agency recently to 8 µg/L [77]. Concentrations of MC-LR used in our study represent rather the upper end of the environmental concentration range occurring in surface waters [4,75]. Despite exceeding environmental concentrations of MCs in this experimental setup, we could not confirm earlier reports of MCs affecting lung cells in vitro [29], even though the model used is capable to take up MCs, presumably via OATPs, and demonstrates a time-dependent increase in protein-MC adducts. Additionally, the initial interest to study cyanotoxin effects on the respiratory tract epithelia originated from epidemiological evidence linking cyanobacterial blooms to adverse respiratory conditions [78,79]. The absence of effects on cell viability and the sub-lethal endpoint of intracellular signaling highlights the importance of the assessment of well-characterized environmental samples or model mixtures to reflect potential co-action and perhaps even synergistic effects of cyanobacterial bloom metabolites beyond MCs. Further, more detailed studies are needed to better characterize concentrations of toxic cyanobacteria and cyanotoxins in aerosols and to more accurately assess and quantify the inhalation exposures and concentrations/doses of cyanotoxins relevant for the airway epithelium. Advanced human in vitro airway epithelium models, such as ALI cultures of immortalized human bronchial epithelial cells, possibly in co-cultures with different cell types [70] shall be considered because they allow for toxicologically more relevant toxin administration in contrast to fully submerged cell cultures. In combination with better estimates of environmental exposures, such an approach would contribute to improved assessment of human health risks associated with inhalation of cyanotoxins.

## 4. Conclusions

To conclude, our study presented similar patterns of MC-LR effects in two respiratory system-relevant human bronchial epithelial cell lines. Both HBE1 and 16HBE14o- cultures transcribed several genes encoding transporting polypeptides, potentially involved in MC-LR cellular uptake. Despite the evidence of both, formation and accumulation of putative MC-LR adducts with intracellular proteins, the cell viability was not significantly compromised by even relatively high (20 µM) MC-LR concentrations. A slight decrease in cell viability induced by the highest toxin concentration in 16HBE14o- cells was associated with an increase in cell impedance, indicating an adaptive response of epithelial cells to sub-cytotoxic concentrations of MC-LR. MC-LR exposure and uptake did not result in any significant alteration of intracellular signal transduction mediated via MAPK ERK1/2 or p38. With respect to the evidence on the toxin uptake and its interactions with intracellular proteins of human bronchial epithelial cells, the effects of MCs in human airway epithelium should be further investigated using physiologically more relevant in vitro models and exposure scenarios.

## 5. Materials and Methods

### 5.1. Cell Cultures

Two non-cancer immortalized human bronchial epithelial (HBE) cell lines were used in the study. HBE1 cells (RRID: CVCL 0287), originating from non-cystic fibrosis lung tissue of a 60-year-old woman, were transfected with human papillomavirus type 18 E6 and E7 oncogenes [80]. HBE1 cells have been reported to exhibit differentiated airway epithelium properties, including polarized phenotype, vectorial ion transport, or expression of cystic fibrosis transmembrane conductance regulator (CFTR) protein [80].

16HBE14o- cells (RRID: CVCL 0112) were obtained by transfection of 40 pSVori^-^ plasmid into 1-year-old human male bronchial epithelial explants [81,82]. The cell line retains physiological and morphological properties of differentiated bronchial epithelial cells, such as contact inhibition, intermediate filaments, polarized ion channels, CFTR protein, as well as tight junctions [82].

Further details of the cell culture work and used materials are given in the Supplementary Materials (Section S1: Methods).

### 5.2. Experimental Design

Stock solutions of MC-LR (100× or 500× concentrated) were prepared in 20% (*v*/*v*) methanol to assure their sterility for the in vitro experiments and stored frozen at −20 °C. Based on preliminary data of cell seeding densities (data not shown), HBE1 and 16HBE14o-cells were seeded at the density of 60,000–80,000 cells/cm^2^ into 96-well black plates for cytotoxicity assays (Greiner, Cat. No. 655090, Greiner BioOne, Kremsmünster, Austria), 96-well E-plates for impedimetric real-time cell analysis (E-Plate VIEW 96 PET, ACEA Biosciences, San Diego, CA, USA) and into Ø 35 mm Petri dishes (Costar, Cambridge, MA, USA) for protein analysis by western blotting and for gene transcription analysis by RT-PCR.

After 24 h of growth in culture medium, cells were exposed to 1–20 µM MC-LR for 48 h for cytotoxicity assessment and 96 h for RTCA, or to 1 µM MC-LR for the assessment by RT-PCR (48-h exposure) and by western blotting after various time intervals (from 1 h up to 48 h). In RTCA and cytotoxicity assays, cells were exposed by replacing the growth medium with MC-LR containing exposure medium. For western blotting analysis, the MC-LR stock solution was pipetted directly into the growth medium to avoid unintentional phosphorylation during medium replacement due to shear forces and growth factor replenishment.

Each experiment was conducted together with negative (untreated) control (NC), and with appropriate solvent control (SC) containing 0.2% (*v*/*v*) (cytotoxicity and impedimetric experiments) or 0.04% (*v*/*v*) (western blotting) methanol.

### 5.3. Impedimetric Real-Time Cell Analysis (RTCA)

The impedimetric RTCA was conducted according to [67]. Briefly, cellular impedance (reported as Delta Cell Index-Fraction of the non-treated Control, DCI-FOC) of both HBE1 and 16HBE14o- cells was detected on xCELLigence SP (ACEA Biosciences, San Diego, CA, USA) as a function of attachment and proliferation. Cell Index (CI) was measured in each well every 15 min during the first 5 h after the cell seeding or exposure start, and every 60 min for the following period up to 96 h. Delta Cell Index values were calculated according to xCelligence System Technical Note No. 2 [83]. For details of the calculation, see Supplementary Materials, Equation S1.

### 5.4. Cytotoxicity Assays

An assay combining three indicative dyes was adopted from Raska et al. [56] to evaluate the disruption of various vital cellular processes: the plasma membrane integrity and esterase activity by 5-carboxyfluorescein diacetate acetoxymethyl ester (CFDA-AM), the metabolic reductive potential as resazurin reduction (Alamar blue^®^ assay), and neutral red uptake (NRU) by lysosomes [48,49]. Further details of the method and used materials are given in the Supplementary Materials (Section S1: Methods).

### 5.5. Reverse-Transcription Polymerase Chain Reaction (RT-PCR)

In the processes of RNA isolation, RT-PCR and agarose gel electrophoresis were conducted according to a recently published study, including the sequences of the primers [56]. Briefly, total RNA was isolated by RNeasy Plus Mini kit (QIAGEN, Hilden, Germany), its concentration and purity were measured by NanoDrop 1000 (ThermoFisher, Prague, Czech Republic). cDNA was prepared using Transcriptor First Strand cDNA Synthesis Kit (Roche, Basel, Switzerland) and followed by PCR reaction (Phusion High-Fidelity DNA Polymerase kit, ThermoFisher) with target-specific primers (sequences given in [56]). HBE1 and 16HBE14o- RNA samples were used in the experiments together with positive controls for OATP genes (HepG2 and HeLa cell lines, liver RNA). PCR was run with the following parameters: initial denaturation for 30 s at 98 °C, 25 cycles of denaturation for 10 s at 98 °C, annealing for 30 s at 60 °C, and elongation for 30 s at 72 °C, the final extension was at 72 °C for 10 min. PCR products were separated by 1.5 % agarose gel electrophoresis, stained by ethidium bromide and detected using MF-ChemiBis 3.2 documentation system with GelCapture software (DNR Bio-Imaging Systems Ltd., Neve Yamin, Israel).

### 5.6. Western Blotting

The western blot analysis was conducted as recently published [56,67]. After exposure, the cells were rinsed 5 times with ice-cold phosphate-buffered saline (PBS), as previously optimized to sufficiently remove residues of extracellular MC-LR (data not shown), to prevent its binding to cellular proteins after the cell lysis and to avoid its interference with the assessment of MC-LR uptake [56]. The proteins were extracted with 150 µL lysis buffer (20 mM Tris base, 1 mM dithiothreitol, 4% (*w*/*v*) sodium dodecyl sulfate, SDS) per dish using a cell scraper. Cell lysates were homogenized by ultrasonication (20 s at 50% power and pulses of 1 s/0.2 s rest; SONOPULS mini20, Bandelin Electronic, Berlin, Germany) and protein concentration measured using DC Protein Assay (Bio-Rad, Hercules, CA, USA). Protein lysates were subsequently diluted to a concentration of 1 mg/mL with lysis buffer and 4× Laemmli sample buffer (Bio-Rad). Proteins were separated by SDS-polyacrylamide gel electrophoresis (SDS-PAGE) on 12.5% acrylamide gels (120 V, 70–90 min) and electrophoretically transferred to polyvinylidene difluoride membrane (100 V, 60 min; Immobilon-P, Merck Millipore, Darmstadt, Germany). Membranes were incubated with blocking solution (5% (*w*/*v*) non-fat dry milk in Tris-buffered saline with 0.1% (*v*/*v*) Tween 20; TBS-T) and subsequently incubated overnight at 4 °C with primary antibodies diluted in blocking solution. After the washing step, membranes were incubated in secondary antibodies for one hour at room temperature. Signals from western blot were detected using ECL Substrate (Bio-Rad) in MF-ChemiBis 3.2 with GelCapture software (DNR Bio Imaging Systems, Neve Yamin, Israel). After quantification of signals, membranes were washed in distilled water and incubated overnight at 4 °C with diluted primary antibody against housekeeping protein GAPDH. Membranes were then incubated in secondary antibody for one hour at room temperature and quantified. The optical density of protein bands was evaluated using ImageJ. Signals from the proteins of interest (i.e., MAPK signals) were normalized to the GAPDH (the loading control) signal from the same sample and blot and to the negative control (NC) from the same blot (for detailed equations see Supplementary Materials, Equation S2). For a list of primary and secondary antibodies used in the study see Supplementary Materials (Section S1: Methods).

### 5.7. Data Evaluation and Statistical Analyses

The data obtained from at least two independently repeated experiments were combined to calculate the mean + standard deviation (S.D.) values, which are presented in the graphs. Initial data evaluation was conducted in MS Excel (Microsoft, Redmond, WA, USA). Data plotting, graphical outputs, and statistical analyses were conducted in SigmaPlot 11.0 (Systat Software Inc., Erkrath, Germany). For data with a normal distribution (Shapiro-Wilk’s test) and equal variances (Equal Variance test), statistical differences from the negative control samples were analyzed by one-way ANOVA followed by Dunn’s or Dunnett’s *post-hoc* tests. Kruskal-Wallis ANOVA on ranks followed by Dunnett’s or Mann–Whitney’s *post-hoc* tests was used for evaluation of data with unequal variances and/or non-normal distribution. *P* values < 0.05 were considered significantly different.

## Figures and Tables

**Table 1:** Respiratory symptoms in mice after administration of microcystin-LR (MC-LR).

MC-LR Administration	Respiratory-Related Symptoms	Reference
Intraperitoneal (acute)	Change in respiratory variables, increased lung impedance, pulmonary lesions, lung inflammation.	Carvalho et al., 2010 [37]; Gupta et al., 2003 [38]; Soares et al., 2007 [39]
Intratracheal instillation (acute)	Increased number alveolar septa collapsed areas, fractured alveolar walls, damage to F-actin, multiple inflammatory-related protein alterations.	Zhao et al., 2018 [40]
Intraperitoneal (20 days)	Impairment of respiratory mechanics, pulmonary parenchyma degradation, augmented contents of inflammatory mediators in lung tissue, a dose-dependent lung inflammatory response.	Carvalho et al., 2016 [41]
Intranasal (30 days)	Impairment of all respiratory mechanical components, pulmonary parenchyma damage marked by the augmented alveolar collapsed areas and the number of inflammatory cells	Oliveira et al., 2015 [42]
Intranasal (30 days)	Lung structure disorder, thickening of alveolar septa, aggregation of inflammatory cells (induction of oxidative stress, altered expression of inflammatory cytokines, etc.)	X. Li et al., 2016 [43]
Inhalation (7 days)	Degeneration and necrosis of nasal respiratory epithelium, neutrophilic inflammation	Benson et al., 2005 [44]

## Supplementary Materials

The following Supplementary Materials are available online at https://www.mdpi.com/2072-6651/12/3/165/s1, Figure S1: Representative images of gene expression in HBE1 and 16HBE14o-cells, Figure S2: Immunodetection of putative MC-LR protein adducts in bronchial cell lines, Figure S3: Effects of 1 µM microcystin-LR on mitogen-activated protein kinase levels in HBE1 cells, Figure S4: Effects of 1 µM microcystin-LR on mitogen-activated protein kinase levels in 16HBE14o- cells; Equation S1: Calculation of Delta-Cell Index-Fraction of Control values from RTCA data, Equation S2: Densitometric calculations for western blot data; Section S1: Methods.
