# Supplementary material for: Microcystin-LR Does Not Alter Cell Survival and Intracellular Signaling in Human Bronchial Epithelial Cells
Source: Toxins (Basel). 2020 Mar 7;12(3):165. doi: 10.3390/toxins12030165 (PMC7150819; doi:10.3390/toxins12030165)
Supplement: Supplementary file 1 [file toxins-12-00165-s001.pdf]

Supplementary Materials: Microcystin-LR Does Not Alter Cell Survival and Intracellular Signaling in Human Bronchial Epithelial Cells

Ondřej Brózman, Barbara Kubickova, Pavel Babica and Petra Laboha

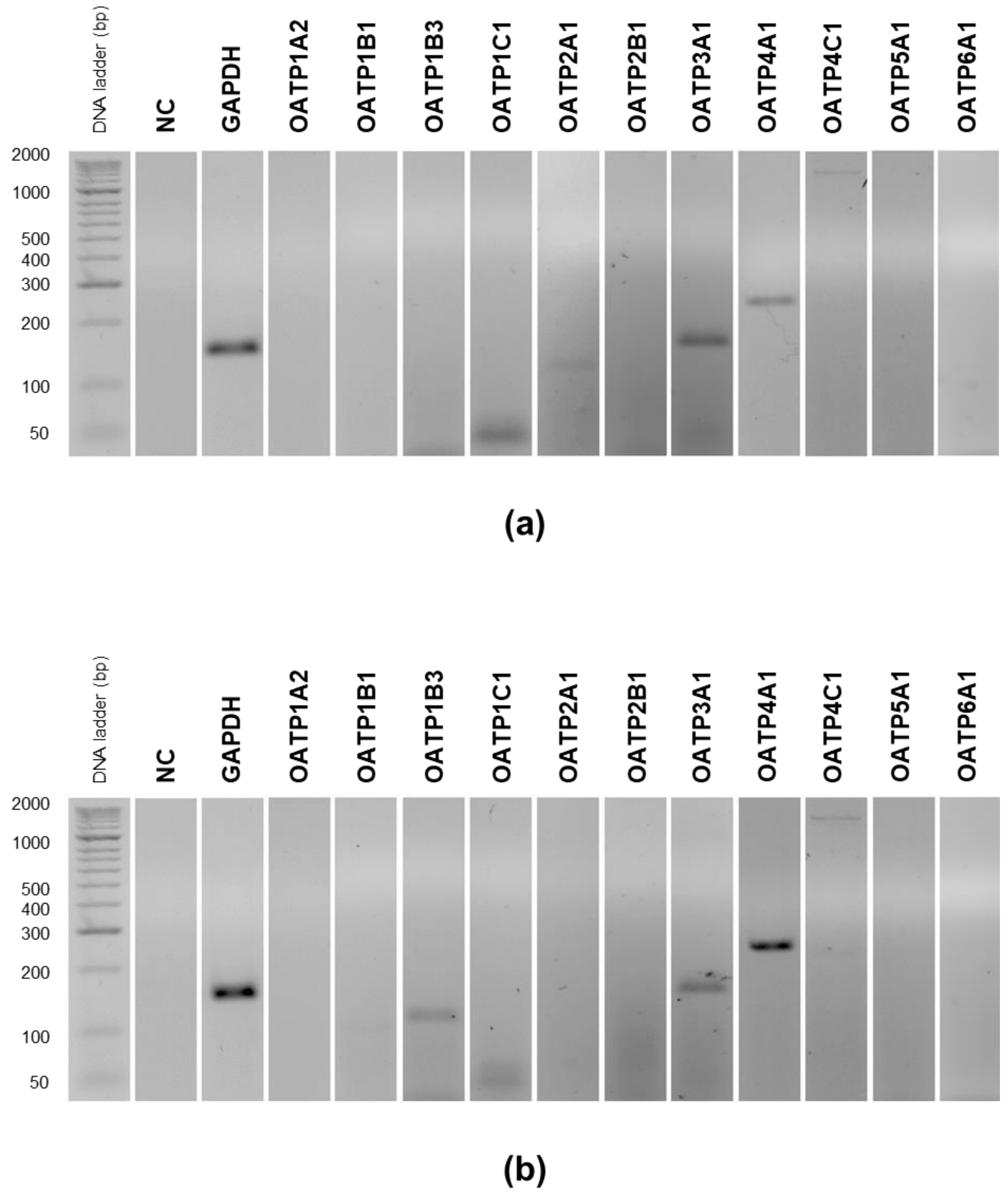

Figure S1. Representative images of gene expression in HBE1 and 16HBE14o- cells. Detection of the expression of genes encoding organic-anion-transporting peptides (OATPs) in HBE1 (**a**) and 16HBE14o- (**b**) cell lines by PCR. NC, negative control; GAPDH, glyceraldehyde-3-phosphate dehydrogenase; OATP, organic-anion-transporting polypeptide.

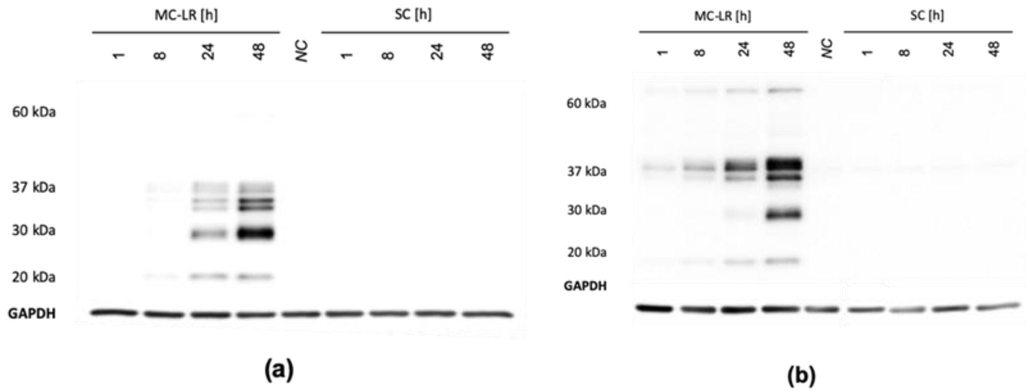

Figure S2. Immunodetection of putative MC-LR protein adducts in bronchial cell lines. Both HBE1 (**a**) and 16HBE14o- (**b**) cell lines were exposed to 1 μM MC-LR for 1, 8, 24 and 48 h. Western blot technique was used to determine the presence of MC-LR protein adducts using the primary anti-MC-LR antibody. Representative blots of independent experiments ($n \geq 2$) are shown. SC, solvent control; MC-LR, microcystin-LR; NC, negative control.

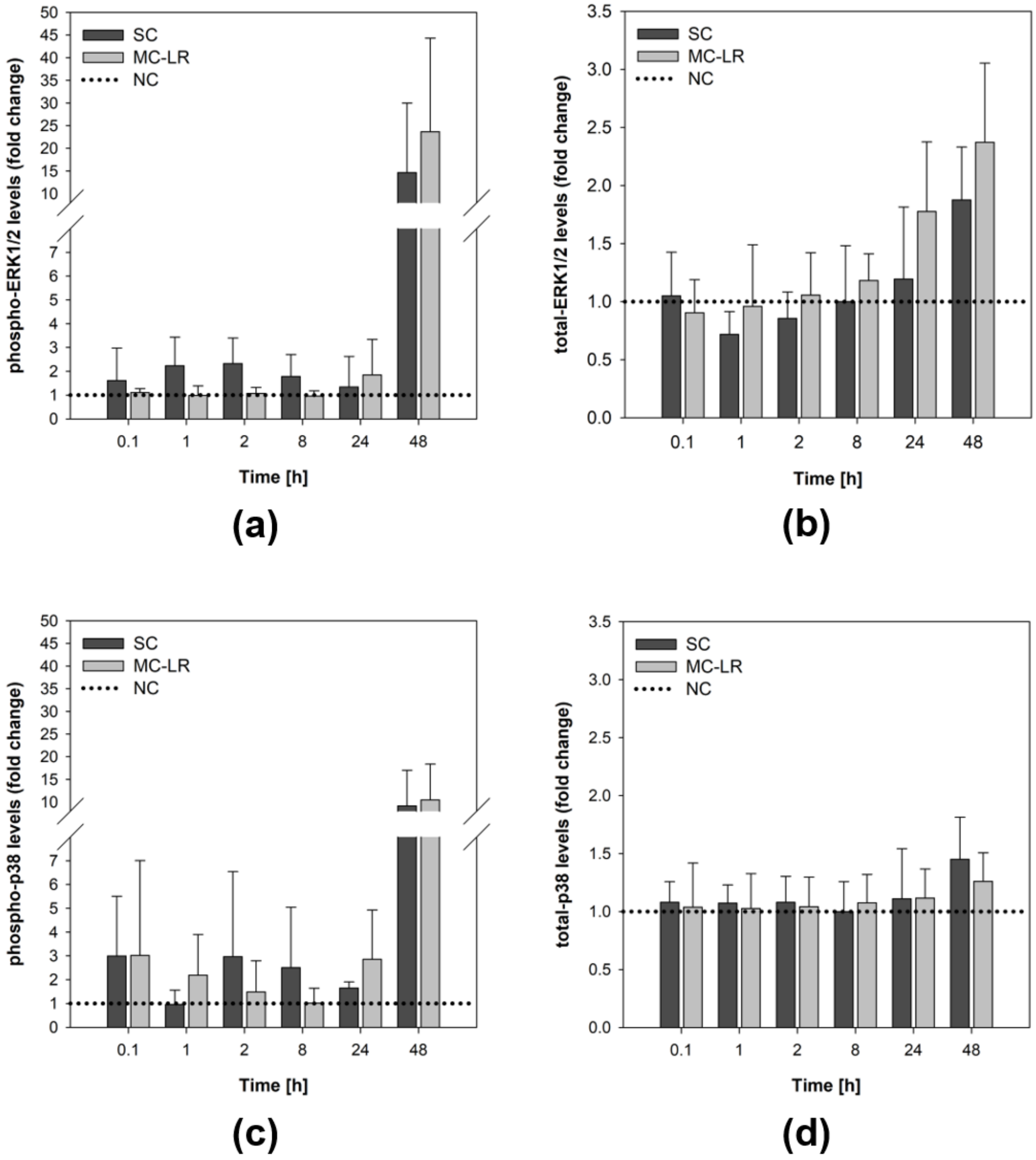

Figure S3. Effects of 1 μM microcystin-LR on mitogen-activated protein kinase levels in HBE1 cells. Results of densitometric evaluation show MAPK levels expressed as fold changes compared to negative control (horizontal dotted line): fold changes in phosphorylated extracellular signal-regulated kinases 1/2 (phospho-ERK1/2; **a**), total ERK1/2 (**b**), phosphorylated p38 kinase (phospho-p38; **c**), and total p38 kinase (**d**). Data were normalized according to equations in Appendix B, Equation B2. Bar charts represent mean + S.D. from independent experiments ($n \geq 2$) in the solvent control (dark grey bars) and MC-LR treatment (light grey bars). SC, solvent control; MC-LR, microcystin-LR; *NC*, negative control, MAPK, mitogen-activated protein kinase.

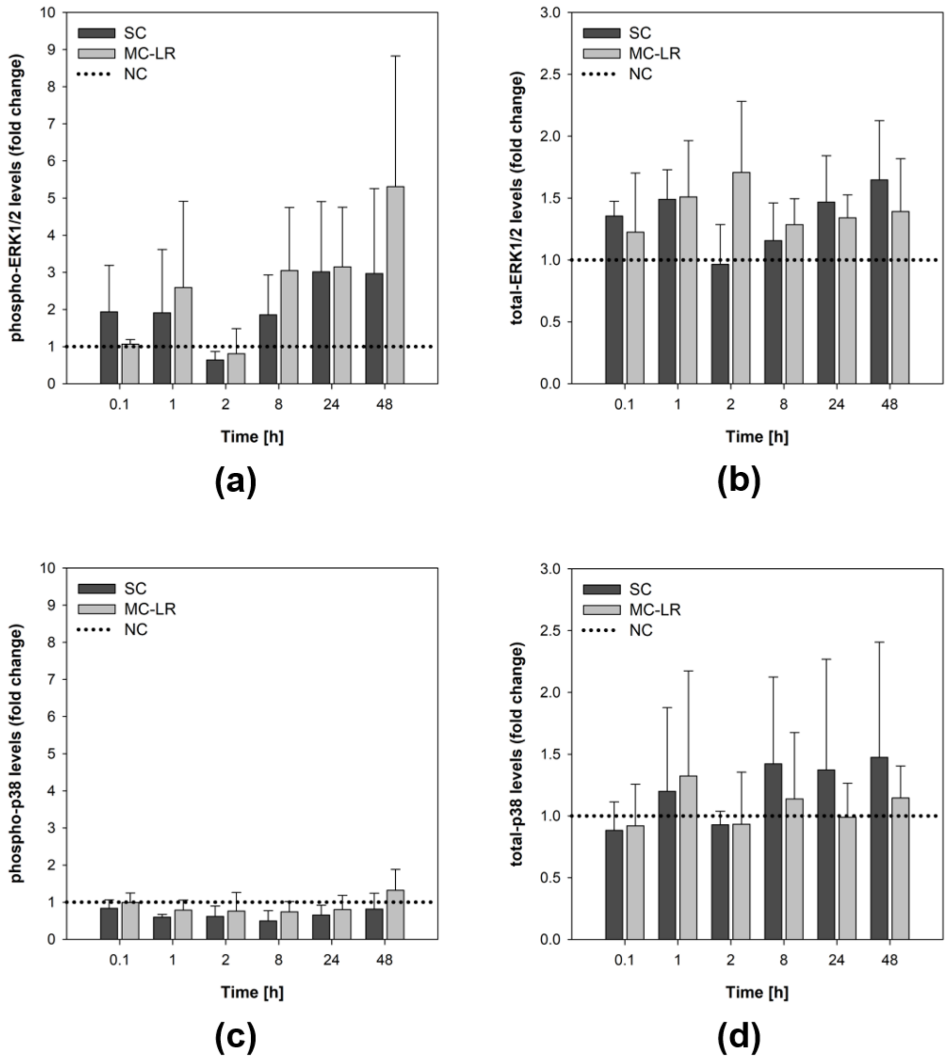

Figure S4. Effects of 1 μM microcystin-LR on mitogen-activated protein kinase levels in 16HBE14o- cells. Results of densitometric evaluation show MAPK levels expressed as fold changes compared to negative control (horizontal dotted line): fold changes in phosphorylated extracellular signal-regulated kinases 1/2 (phospho-ERK1/2; **a**), total ERK1/2 (**b**), phosphorylated p38 kinase (phospho-p38; **c**), and total p38 kinase (**d**). Data were normalized according to equations in Appendix B, Equation B2. Bar charts represent mean + S.D. from independent experiments ($n \geq 2$) in the solvent control (dark grey bars) and MC-LR treatment (light grey bars). SC, solvent control; MC-LR, microcystin-LR; NC, negative control, MAPK, mitogen-activated protein kinase.

$$\text{DCI-FOC}_{well}(t_x) = \frac{\text{DCI}_{treated}}{\text{DCI}_{control}} = \frac{\text{CI}_{treated}(t_x) - \text{CI}_{treated}(t_{Delta}) + \text{Delta Constant}_{CI\_Plate\_max}(t_{Delta})}{\text{CI}_{control}(t_x) - \text{CI}_{control}(t_{Delta}) + \text{Delta Constant}_{CI\_Plate\_max}(t_{Delta})}$$

Equation S1. Calculation of Delta-Cell Index-Fraction of Control values from RTCA data. Delta-Cell Index-Fraction of the non-treated (negative) Control (DCI-FOC) was calculated, where Delta Time (t_{Delta}) corresponds to the last Cell Index (CI) readout prior the initiation of the exposure (i.e. 24 h post-seeding), Delta Constant corresponds to the maximum CI readout from the Plate (from all control and experimental wells) at the Delta Time.

$$(a)\ \boldsymbol{MAPK\ relative\ density\ (levels)\ after\ MC\text{–}\ LR\ treatment\ (fold\ change)} = \frac{\boldsymbol{MAPK\ (GAPDH\_normalized)_{MC-LR,\,t=x}}}{\boldsymbol{MAPK\ (GAPDH\_normalized_{NC,\,t=1}}} = \frac{\frac{\boldsymbol{MAPK_{MC-LR,\,t=x}}}{\boldsymbol{GAPDH_{MC-LR,\,t=x}}}}{\frac{\boldsymbol{MAPK_{NC,\,t=1}}}{\boldsymbol{GAPDH_{NC,\,t=1}}}}$$

$$(b)\ \boldsymbol{MAPK\ relative\ density\ (levels)\ in\ solvent\ control\ (fold\ change)} = \frac{\boldsymbol{MAPK\ (GAPDH\_normalized)_{SC,\,t=x}}}{\boldsymbol{MAPK\ (GAPDH\_normalized_{NC,\,t=1}}} = \frac{\frac{\boldsymbol{MAPK_{SC,\,t=x}}}{\boldsymbol{GAPDH_{SC,\,t=x}}}}{\frac{\boldsymbol{MAPK_{NC,\,t=1}}}{\boldsymbol{GAPDH_{NC,\,t=1}}}}$$

$$(c)\ \boldsymbol{MAPK\ normalization\ to\ solvent\ control,\ MC\text{–}\ LR/SC\ ratio\ (fold\ change)} = \frac{\frac{\boldsymbol{MAPK\ (GAPDH\_normalized)_{MC-LR,\,t=x}}}{\boldsymbol{MAPK\ (GAPDH\_normalized_{NC,\,t=1}}}}{\frac{\boldsymbol{MAPK\ (GAPDH\_normalized)_{SC,\,t=x}}}{\boldsymbol{MAPK\ (GAPDH\_normalized_{NC,\,t=1}}}}$$

Equation S2. Densitometric calculations for western blot data. Equations: MAPK levels (relative density) after MC-LR treatment expressed as fold change over negative control (**a**), MAPK levels in the solvent control expressed as fold change over negative control (**b**), MAPK MC-LR/SC ratio comparing MAPK levels after MC-LR treatment versus solvent control (**c**). *NC*, negative control; *SC*, solvent control; *MC-LR*, microcystin-LR; *P-ERK1/2*, phosphorylated extracellular signal-regulated kinases 1/2; *t-ERK1/2*, total extracellular signal-regulated kinases 1/2; *P-p38*, phosphorylated p38 kinase; *t-p38*, total p38 kinase; *GAPDH*, glyceraldehyde-3-phosphate dehydrogenase.

1. Methods

1.1. Cultivation of HBE1 Cells

If not stated otherwise, all following materials and supplements were purchased from Sigma Aldrich (Prague, Czech Republic). Cells were grown in a phenol red containing DMEM/F12 growth medium (Biowest, L0095, Nuaillé, France), further supplemented with 4 μg/mL human insulin, 5 μg/mL transferrin, 10 ng/mL human epidermal growth factor, 0.1 μM dexamethasone, 20 ng/mL cholera toxin, 2.5 μg/mL Plasmocin™ Treatment (InvivoGen, San Diego, CA, USA), 2.5 mM L-glutamine and 0.1% (*v*/*v*) endothelial cell growth supplement (PromoCell, Heidelberg, Germany). After addition of all the supplements, pH was adjusted to 7.40 ± 0.05 and medium sterile-filtered using 0.2 μm top-bottle polyethersulfone filter (TPP, Trasadingen, Switzerland) into sterile glass bottles. Cells were routinely cultured in culture flasks suitable for adherent cell lines (TPP) in incubator with standard conditions for in vitro mammalian cell cultivation

(humified 5 % CO_2 atmosphere at 37 °C). Cells between passages 25–30 were used for the experiments to avoid variations.

1.2. Cultivation of 16HBE14o-Cells

The cells were routinely cultured in phenol red-containing MEM medium (Gibco, 61100, ThermoFisher Scientific, Prague, Czech Republic), supplemented with 2 mg/L sodium bicarbonate (Lach-ner, Neratovice, Czech Republic) with pH adjusted to 7.40 ± 0.05. The cell medium was sterile-filtered using 0.2 µm top-bottle polyethersulfone filter (TPP) into sterile glass bottles and supplemented with 10% (*v*/*v*) fetal bovine serum (South America, Biosera, Prague, Czech Republic). Cells were routinely cultured in culture flasks suitable for adherent cell lines (TPP) in incubator (Shel Lab, Cornelius, OR, USA) with standard conditions for in vitro mammalian cell cultivation (humified 5% CO_2 atmosphere at 37 °C). Cells between passages 113–117 were used for the experiments to avoid variations.

1.3. Cytotoxicity Assay

Cells were washed with PBS and incubated for 30 min in 100 µL phenol red-free DMEM/F12 (Sigma Aldrich, D6434) medium with 5% (*v*/*v*) AlamarBlue® (ThermoFisher Scientific, Pardubice, Czech Republic) and 4 µM 5-carboxyfluorescein diacetate, acetoxymethyl ester (CFDA-AM; C1354, ThermoFisher Scientific). Next, cells were analysed using FLUOstar Optima (BMG Labtech, Prague, Czech Republic) for fluorescence at 530/590 nm (ex/em) and 485/520 nm (ex/em) respectively. Subsequently, cells were rinsed three times with PBS and incubated in 100 µL phenol red-free DMEM/F12 medium with 50 µg/mL Neutral red solution (Sigma Aldrich) for 90 min. Afterwards, cells were rinsed with PBS and neutral red accumulated by the viable cells was lysed by 1% (*v*/*v*) acetic acid in 50% (*v*/*v*) EtOH (Lach-ner). NRU was evaluated by measurement of absorbance using POLARstar Optima (BMG Labtech) at 540 nm and a reference wavelength 690 nm. The obtained results from experimental treatment were compared to non-treated NC and expressed as percentage of viability.

1.4. Primary and Secondary Antibodies for Western Blotting

All primary and secondary antibodies were purchased from Cell Signalling Technology (Danvers, MA), if not specified otherwise.

The following primary antibodies and dilution factors were used—1:1000 diluted polyclonal rabbit antibody Phospho-p44/42 MAPK ERK1/2 (#4370S), Phospho-p38 MAPK (#4511S), p44/42 MAPK ERK1/2 (#4695), p38 MAPK (#8690S), 1:2000 diluted monoclonal mouse antibody against glyceraldehyde-3-phosphate dehydrogenase (GAPDH; Merck Millipore, Prague, Czech Republic, #MAB374), 1:500 diluted monoclonal mouse antibody against Microcystin-LR (MC10E7; Alexis, Lausen, Switzerland, #ALX-804-320-C200).

The following secondary antibodies and dilution factors were used—1:2500 diluted goat polyclonal horseradish peroxidase-conjugated Anti-Rabbit IgG (#7074S), 1:2500 diluted horse polyclonal horseradish peroxidase-conjugated Anti-Mouse IgG (#7076S).
